# Formation of net-like patterns of gold nanoparticles in liquid crystal matrix at the air–water interface

**DOI:** 10.1007/s11051-012-0826-4

**Published:** 2012-03-31

**Authors:** Jan Paczesny, Krzysztof Sozański, Igor Dzięcielewski, Andrzej Żywociński, Robert Hołyst

**Affiliations:** 1 Institute of Physical Chemistry, Polish Academy of Sciences, Kasprzaka 44/52, 01-224 Warsaw, Poland; 2 Institute of High Pressure Physics Unipress, Polish Academy of Sciences, Sokołowska 29/37, 01-142 Warsaw, Poland

**Keywords:** Gold nanoparticles, Langmuir films, Langmuir–Blodgett films, Nanostructures, Net, Nanoporosity

## Abstract

Controlled patterning and formation of nanostructures on surfaces based on self-assembly is a promising area in the field of “bottom-up” nanomaterial engineering. We report formation of net-like structures of gold nanoparticles (Au NPs) in a matrix of liquid crystalline amphiphile 4′-*n*-octyl-4-cyanobiphenyl at the air–water interface. After initial compression to at least 18 mN m^−1^, decompression of a Langmuir film of a mixture containing both components results in formation of net-like structures. The average size of a unit cell of the net is easily adjustable by changing the surface pressure during the decompression of the film. The net-like patterns of different, desired average unit cell areas were transferred onto solid substrates (Langmuir–Blodgett method) and investigated with scanning electron microscopy and X-ray reflectivity (XRR). Uniform coverage over large areas was proved. XRR data revealed lifting of the Au NPs from the surface during the formation of the film. A molecular mechanism of formation of the net-like structures is discussed.

**Electronic supplementary material:**

The online version of this article (doi:10.1007/s11051-012-0826-4) contains supplementary material, which is available to authorized users.

## Introduction

“Bottom-up” lithography, also denoted as “soft” lithography, has been recognized as complementary to the widely used “top-down” approach (Moriarty 2001). Nano- and atomic-scale manipulation by scanning probe techniques as well as self-assembly can possibly overcome some of the limitations of classical methods (Whitesides et al. 1991; Kiely et al. 1998; Li et al. 1999; Pileni 2001; Fendler 2001; Sanchez et al. 2001). A properly planned and managed sequence of steps of “bottom-up” self-assembly may allow obtaining complex systems. “Top-down” is gradually approaching its limits, as device features are down-scaled into the sub-100-nm regime. The cost of miniaturization associated with lithography equipment and operating facilities is likely to create an economical barrier for development of conventional processors and memory chips (Frank et al. 2001; Likharev 2003). It seems that the ideal way of preparation of future electronic devices would be one combining these two approaches, i.e., facilitating a scaffold, obtained via lithography or other high-precision technique, decorated with a functional pattern of precisely located nanoscale objects (Bjørnholm et al. 1998). Selective, controlled patterning of surfaces with nanoscale objects is therefore a matter of significant importance and much effort has to be directed to understanding forces governing the self-assembly processes (Bjørnholm et al. 1999).

Among different techniques applicable for “bottom-up” material fabrication, Langmuir–Blodgett (LB) deposition has been found to be one of the most promising. It requires simple equipment, yet ensures high reproducibility and very good control over the process and the prepared structures. Moreover, a variety of chemical building blocks for self-assembly is available. An ordered monolayer can be easily transferred onto solid substrates with great fidelity (Blodgett and Langmuir 1937).

One of very important topics in contemporary research on thin films is films of periodically organized nanoporosity (Sanchez et al. 2008). Cellular structures were obtained in case of Au NPs (Kane et al. 2010) as well as Ag NPs (Sun et al. 2001) or Co NPs (Petit et al. 1998). One- and two-dimensional networks of nanoparticles are among the most applicable ones (Srivastava and Kotov 2009). The authors described the possible applications of unique optical (spectroscopy), magnetic (bio-imaging), and electronic (single electron transistors) properties of such structures. 1D array of Pd NPs was used to manufacture a gas sensor (Favier et al. 2001). In another example, a DNA detection sensor was based on a 2D structure of Au NPs (Charrier et al. 2006). An interesting paper by Kane et al. (2010) describes an Au NPs net which exhibits electronic switching based on gating by metabolic activity of yeast cells deposited on the structure.

Net-like structures were also found in LB systems, where gold nanoparticles are mixed with amphiphilic molecules such as polymers (Hansen et al. 2008) or phospholipids (Hassenkam et al. 2002; Mogilevsky et al. 2010). Hassenkam et al. (2002) described a mechanism of formation of a net-like structure by hydrophobic Au NPs. The main idea is that the nanoparticles act as impurities, which are repulsed to the edges of domains solidifying during compression of the Langmuir films. Mogilevsky et al. (2010) confirmed such explanation by systematic studies on behavior of Au NPs in LB films of a number of phospholipids.

Some mixtures of Au NPs and liquid crystals have already been studied. For instance, Vijayaraghavan and Kumar (2009) described an ordered structure of Au NPs in a matrix of a discotic LC. Our previous work concerning bolaamphiphiles focused on preparation of surfaces of desired extent of coverage by an ordered, densely packed monomolecular film (Paczesny et al. 2011). The liquid crystals were recognized as an ideal template for the “bottom-up” approach of preparation of more complex nanostructures. The recent review by Bisoyi and Kumar (2011) concerning LC as an emerging avenue of soft self-assembly refers to around ninety articles showing possibilities of utilization of LC properties.

We used the LB technique to create and transfer onto a solid substrate the net-like structure of controlled morphology based on amphiphilic gold nanoparticles and 4′-*n*-octyl-4-cyanobiphenyl (8CB) mixtures.

Pressure-induced layering transitions of 8CB have been already intensively studied by means of: ellipsometry (Xue et al. 1992), Brewster angle microscopy (BAM) (de Mul and Mann 1994, 1998; Friedenberg et al. 1994; Modlińska et al. 2009), surface potential measurements (Schmitz and Gruler 1995), and optical second harmonic generation (SHG) (Guyot-Sionnest et al. 1986). The SHG technique was also used to confirm spontaneous organization of 8CB molecules evaporated onto a solid substrate into multilayer stacks (Olenik et al. 2003). Thus behavior of 8CB in LB systems is well known and described.

Behind the collapse point 8CB does not form disordered aggregates, yet a process of formation of a multilayer occurs. The plateau region of the π–*A* isotherm corresponds to formation of liquid domains of a trilayer film. At the end of this plateau, the entire film is a trilayer stack. Upon further compression a rise of surface pressure is observed. The second collapse point corresponds to a very close packed trilayer film. Further decrease of available area leads to a break in the film and formation of even thicker multilayer stacks (de Mul and Mann 1994, 1998; Friedenberg et al. 1994).

## Experimental

### Materials

8CB was obtained from Merck and used as received to prepare a fairly dilute (1.04 mg cm^−3^) solution in high purity (spectrally clean) chloroform. Gold nanoparticles (Au NPs) were synthesized according to a procedure described elsewhere (Jana and Peng 2003) and subsequently functionalized with TMA, so that ca. 10% of original undecanethiol ligands were substituted for TMA (Kalsin et al. 2006). Mean diameter of Au NPs, assessed by means of SAXS measurements and AFM images analysis, was 8.9 nm. The size distribution was estimated to be less than 10% based on high resolution scanning electron microscopy (SEM) pictures. Au NPs exhibited a single, moderately narrow peak of zeta potential at +35 mV. For the analytical data on Au NPs, see Supporting Information. In all the experiments, 1:1 chloroform/methanol solution of concentration of 0.5 mg(Au) ml^−1^ was used. The amphiphilic Au NPs were found to form stable Langmuir films. Upon spreading at the air–water surface, the thiols rearrange so that the polar groups are in contact with the water surface (Nørgaard et al. 2004).

### Methods

The LB experiments were performed with use of a 5 × 75 cm Langmuir trough (NIMA, Coventry, England), enclosed in an acrylic glass cabinet. The system was equipped with surface pressure and potential sensors, a dipper and a temperature control unit, as well as an NFT MiniBAM Brewster angle microscope. Millipore filtered water (18.2 MΩ cm) was used as a subphase. To ensure reproducibility of achieved results, prior to every experiment the trough was carefully cleaned with ethanol and rinsed with water. Any remaining impurities floating on the subphase were removed in iterated process of putting the barriers together as close as possible and removing the surface layer of water from in-between the barriers with an aspirator. Rather than a traditional Wilhelmy plate, single-use filter paper stripes were utilized in the surface pressure sensor. It was calibrated before every experiment.

Solutions were applied onto the subphase with a Hamilton microsyringe. The mixtures of two components (8CB and Au NPs) were prepared beforehand. After 15 min needed for the solvent to evaporate, films were compressed at a constant rate of 10 cm^2^ min^−1^. BAM images were procured in situ, whereas for SEM and X-ray reflectivity (XRR) measurements films had to be transferred onto a solid substrate. The material of choice was polished silicon of very low roughness, cut into 2.5 × 0.8 cm wafers, purchased from the Institute of Electronic Materials Technology, Warsaw, Poland. After cleaning with acetone and treating for at least half an hour with ca. 30% nitric acid, wafers were rinsed with water. The wafer was submerged vertically in the trough prior to applying the investigated solution onto the water surface. Film deposition was conducted according to the LB technique at a dip speed of 5 mm min^−1^, always during an upstroke.

XRR measurements were performed on a Bruker D8 Discover diffractometer, operating at a wavelength of 1.54 Å, with monochromatic parallel beam formation by a parabolic Goebel mirror. The system was equipped with an Eulerian cradle and a reflectometry sample-stage, which ensured precise sample positioning. Scintillation counter together with an automatic absorber of primary beam allowed for linear dynamic range better than 10^8^ cps. For data analysis and fitting, Leptos 4.02 software was applied.

SEM images were taken with use of a Zeiss LEO 1530 scanning electron microscope in the InLens detection mode.

## Results and discussion

A study of thin films of mixtures of Au NPs and 8CB is hereby presented. For the sake of convenience, concentrations of solutions used in LB experiments are expressed in terms of volume of the solution needed to cover 1 cm^2^ of the surface with a dense monolayer (for more details see Supporting Information). The ratio of such concentrations equals in fact the fraction of surface covered by one of the mixture components. The reason why we use such system is that it makes it easy to follow the evolution of patterns with changes of the Au NPs surface coverage. For example, a ratio of 1:1 means that both components cover the same area at the interface when a monomolecular film is formed. A ratio 1:27 means that the area covered by 8CB is 27 times greater than by Au NPs.

**Fig. 1:**
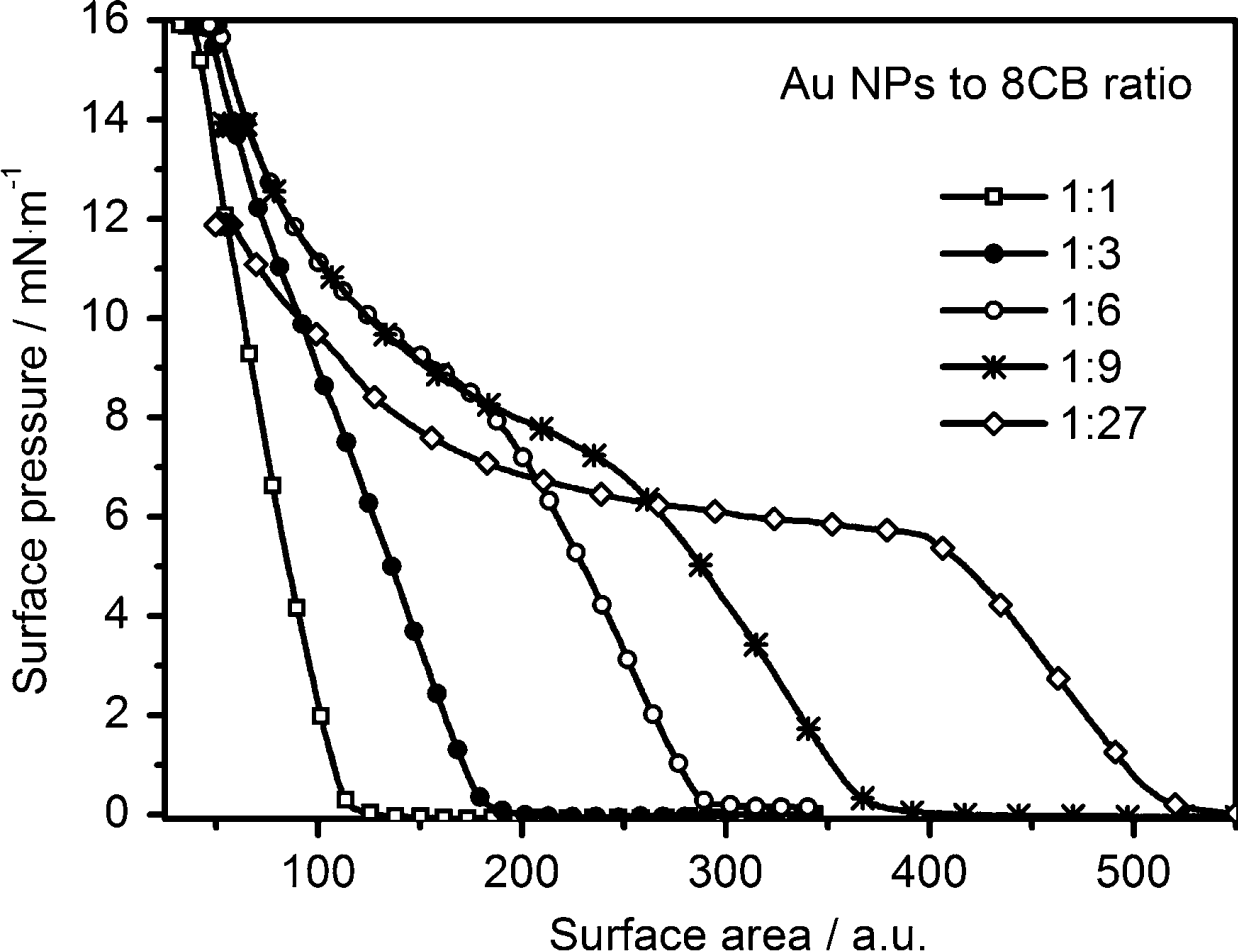
π–*A* isotherms of mixtures of Au NPs and 8CB of different composition. The *x*-axis was scaled so that the amount of Au NPs per an area unit was the same in all the plots. Only the amount of 8CB changed

The influence of changes of the ratio of the components on the obtained π–*A* isotherm was investigated. The results are shown in Fig. 1. The isotherms are scaled with respect to the area occupied by Au NPs. We assumed that the diameter of single Au NPs did not change significantly during the compression–decompression process and the surface coverage ratio is only influenced by the composition of the mixture spread on the water surface. Therefore the scaling, based only on the area occupied by both 8CB and Au NPs, was linear. With changes of proportions of the two mixture components, the shape of the isotherm evolved from one characteristic for pure Au NPs towards the shape obtained for pure 8CB. The isotherm of a mixture of 1:27 ratio had a characteristic plateau, which corresponded to the formation of a trilayer film of 8CB. In case of the isotherm of a 1:1 sample, the character of the curve was predominantly determined by the compression of Au NPs (for an isotherm of pure Au NPs see Fig. S5 in Supporting Information).

**Fig. 2:**
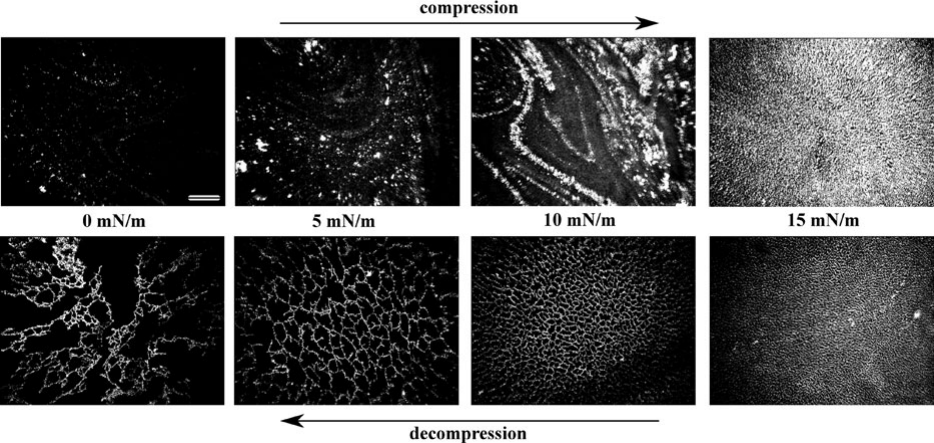
BAM pictures of a film of a mixture of Au NPs–8CB of composition 1:9. The net-like structure was visible only during the decompression. *Scale bar*: 500 μm

BAM images of the film were captured in real time during the compression-decompression experiments. In general, the brighter domains observed in the BAM images corresponded to the more reflective regions, populated by the Au NPs (Hoenig and Moebius 1991). Exemplary BAM pictures taken during compression-decompression cycle of a film of a 1:9 mixture are shown in Fig. 2. The morphology observed during compression did not differ from the one observed when only Au NPs were spread at the air–water interface (for comparison see Fig. S6 in Supporting Information). The pictures shown in the bottom row of Fig. 2 revealed however the formation of the net-like structure of Au NPs in 8CB matrix during decompression. The film was initially compressed up to at least 18 mN m^−1^ and then immediately decompressed. The net-like structure was visible in BAM during the decompression starting from surface pressure of around 15 mN m^−1^. It was not visible in range of surface pressure from 18 to 15 mN m^−1^ due to the limited resolution of the used equipment. Very similar patterns featuring far smaller net unit cells were observed with use of SEM in samples transferred onto a solid substrate at surface pressures up to 17 mN m^−1^, however, still only in case of films that were initially compressed to higher values of surface pressure.

**Fig. 3:**
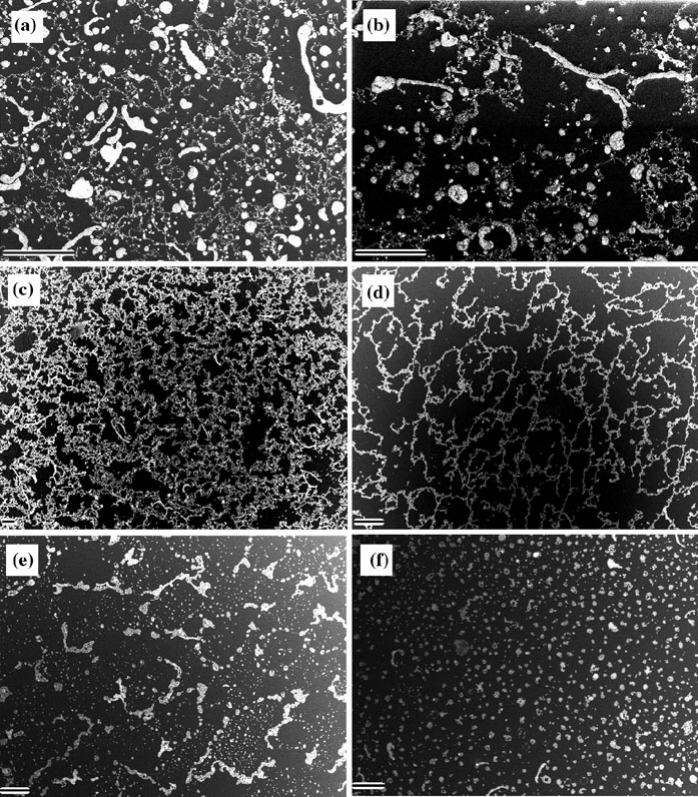
SEM pictures of films transferred at 15 mN m^−1^ of Au NPs–8CB mixtures of different compositions: **a** 1:1, **b** 1:3, **c** 1:6, **d** 1:9, **e** 1:27 initially compressed to 18 mN m^−1^, and **f** 1:9 without initial compression and no time interval before the transfer. *Scale bar*: 20 μm

The SEM images of films of mixtures of different compositions are presented in Fig. 3. The films were transferred according to the LB method, after initial compression to 18 mN m^−1^ and decompression to surface pressure of 15 mN m^−1^. Then the transfer was started after a time interval of 3 min. SEM images of films prepared from mixtures of composition ratio 1:6 (Fig. 3c) and 1:9 (Fig. 3d) reveal Au NPs net-like structures of very similar morphology as that observed with use of BAM. As the amount of Au NPs in the mixture was increased, aggregates started to appear and the structure was strongly deformed (Fig. 3a, b). On the other hand, in case of a film of 1:27 composition (small amount of Au NPs), the net was not complete, i.e., the net units were open and not well marked (Fig. 3e).

SEM picture of a film transferred at 15 mN m^−1^ without the initial compression step and no time interval is shown in Fig. 3f. No net-like structure was detected, which was in line with the BAM observations. We assumed that such film corresponded to the structure of the film observed with use of BAM during compression (verify with Fig. 2). Therefore, it can be concluded that the net-like structure was not present during the compression step up to ~18 mN m^−1^.

In case of a film transferred immediately after reaching the target surface pressure of 18 mN m^−1^, the Au NPs were relatively densely distributed at the surface. It was difficult to determine whether the net-like structure was present. If this was the case, the size of a unit cell of the net was very small—in the range of dimensions of the net “frames” composed of Au NPs (see Fig. S7 in Supporting Information), so that any hypothetical structure would be practically undistinguishable.

Presence of organic compounds (8CB and thiols at the Au NPs surface) might be undesirable for further utilization of the obtained surfaces. Therefore, we dipped the samples in the NaBH_4_ solution for 2 h. Such treatment is known to reduce the S–Au bonds (Yuan et al. 2008). This procedure resulted in a bare gold surface without any organic residues and the net-like patterns were preserved (See Fig. S8 in Supporting Information).

**Fig. 4:**
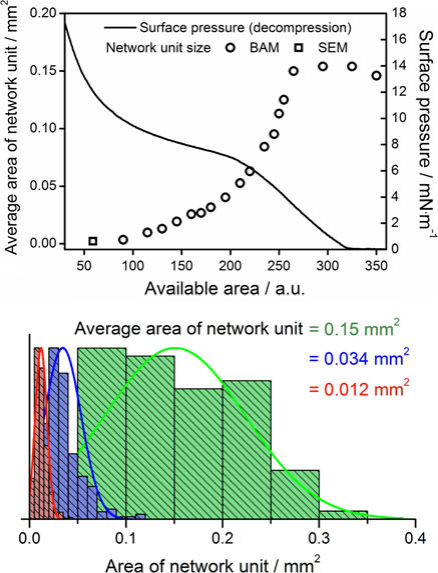
Average size of a unit of a net-like structure versus area of the water subphase upon decompression from 18 mN m^−1^of a film of mixture of composition 1:9. Size distribution histograms revealed increased polydispersity of the average area of a unit cell upon decompression

During the decompression process, the area of the unit cell of the net-like structure increased, as can be noticed in Fig. 2. Therefore, the morphology could be controlled not only by means of the composition ratio of the 8CB–Au NPs mixtures. Also the surface pressure during the decompression and available area of the subphase influenced the film. We determined the average area of the net unit cell within the film of composition ratio 1:9 at different points during the decompression from 18 mN m^−1^. The decompression started just after reaching the target surface pressure, and the barriers moved at a speed of 4 cm^2^/min (the slowest possible). The result of analysis of unit cell size correlated with the π–*A* decompression curve is shown in Fig. 4. The first point at the plot was based on the analysis of SEM pictures of a film transferred onto solid substrate at 15 mN m^−1^. Further analysis was based on BAM images. The increase of the average size of a unit cell could be stopped at a desired value by fixing the movable barriers’ positions.

When evolution of a single cell is concerned, it can be noticed that in the beginning of the decompression process it grows and the “frames” are thinning. At some point the net units begin to merge. There is a limit of the expansion of the structure. The histograms show a significant increase of polydispersity of the unit cells upon decompression.

**Fig. 5:**
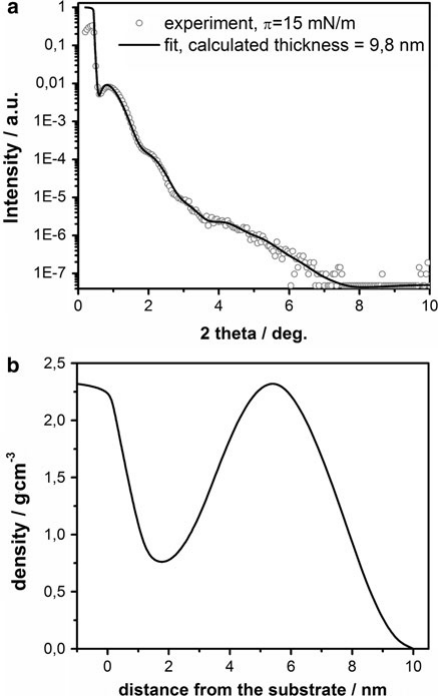
**a** *Open circles* XRR profile of sample of mixture composition 1:9 transferred at 15 mN m^−1^; *solid curve* simulated fit curve, **b** density profile used for XRR pattern simulation corresponded to Au NPs located on the top of an 8CB monolayer

XRR measurements are very sensitive for probing film thickness, roughness and density variations in the direction perpendicular to the surface. The method is, therefore, very suitable for thin film investigation. Its disadvantage is that the data simulation is nonunique in the sense that a change of one parameter can be compensated by other subsequent changes throughout the model structure. Therefore it is crucial to set the starting parameters within physically reasonable values or determine them by means of other techniques. We used the CPK model (purely geometrical model named from the names of Robert Corey, Linus Pauling, and Walter Koltun) for determination of the simulation starting parameters based on the dimensions of 8CB and the organic shell of the Au NPs. We kept the radius of the metallic core of the gold nanoparticles constant and equal to the value obtained from SAXS measurements (whole nanoparticle radius) minus the length of the dodecanothiol molecule (organic shell). The starting parameters could vary only in a reasonable, restricted range (±10%) with exception for the thickness of the sublayer of the metallic core of Au NPs. The results of XRR measurements for a sample of composition ratio 1:9, transferred at 15 mN m^−1^, are shown in Fig. 5. We tested three different models of possible molecular arrangement of the net-like structure: (1) the Au NPs were in contact with the surface of the substrate, (2) the Au NPs were placed on the top of a monolayer of 8CB, (3) the Au NPs were placed on the top of a trilayer of 8CB.

The best fit was obtained for a model that assumed Au NPs placed on top of a monolayer of 8CB. The film thickness (9.8 nm) was noticeably greater than the dimensions of the nanoparticles (8.9 nm). The maximum density of the metallic core sublayer was found at a distance of 5.4 nm from the substrate. It might be assumed that this is where, on average, the centers of the Au NPs were located. Therefore, the distance from 5.5 to 9.8 nm from the substrate surface (i.e., 4.3 nm) corresponds to the radius of an Au NP. According to SAXS measurement, this value equals 4.45 nm (half of the diameter). Thus, the thickness of the layer placed underneath the Au NPs was around 1.2 nm. In a fully extended conformation, the 8CB molecule is around 1.8 nm long. Therefore some interdigitation of aliphatic chains of 8CB and organic shells of Au NPs must have been observed.

The lifting of Au NPs during the net-like structures formation was observed previously by Hansen et al. (2008). This observation will be considered in more detail later on in this article as a part of the discussion on the mechanism of formation of the net-like structures.

Moreover, we performed detailed studies on the distribution of the nanoparticles within a composite film. XRR is an optimal technique for such investigations due to its sensitivity to electron (and hence mass) density variations perpendicular to the solid substrate. As a result of fitting of XRR patterns, the density of the Au NPs sublayer was obtained (Ruiz et al. 2005). In case of a perfect coverage of the surface with gold, this value should be equal to the density of gold. The value of the simulated density of the gold sublayer corresponded to the decrease of the coverage of the surface with Au NPs. Influence of the presence of the organic moieties at the same *z*-distance from the substrate as the metallic core on the density was neglected. The fitted value of density of the Au NPs sublayer (2.3 g cm^−3^) was compared with the expected density for a single layer of Au NPs. The value 2.3 g cm^−3^ indicated that 12% of the surface was covered with Au NPs. The coverage of the surface with Au NPs estimated from the SEM image (see Fig. 3d) was around 13.5 % (based on the image analysis). The results obtained with use of SEM and fitting of XRR patterns were in nearly perfect agreement. This conformity and the very good quality of the fit give great confidence in obtained simulated parameters.

### Mechanism of net-like structures formation

In case of relatively small amounts of Au NPs incorporated within the 8CB matrix analysis of the π–*A* isotherms indicated no significant alterations of compression properties and phase transitions of 8CB. Only in such case, the well-ordered net-like structure was formed. Increased amount of the Au NPs in the mixture led to a disordered film with large aggregates, whereas too small amount of Au NPs was insufficient to form a complete network.

The gold nanoparticles act as impurities dissolved in a 2D solvent. The limited solubility of the particles in the LC film may cause diffusion-limited growth of a fractal, net-like structure (Jensen 1999; Taylor et al. 2001). Similar net-like structures were previously found in phospholipids and polymer matrices at the air–water interface (Hansen et al. 2008; Hassenkam et al. 2002; Mogilevsky et al. 2010). The model proposed by Hassenkam et al. (2002) was found to work in case of hydrophobic Au NPs. The authors did not observe any structure of this kind in case of mixtures of surfactants and Au NPs of increased hydrophilicity. It was explained in terms of competition of interactions between Au NPs themselves and Au NPs and the water surface. For instance, the hydrophobic Au NPs do not interact strongly with the water surface, and therefore can be lifted and “frozen out” from the domains of the condensed phase. Lifting was confirmed by means of XRR measurements (Hansen et al. 2008). The increased hydrophilicity of the Au NPs resulted in different morphology of the film, because the Au NPs should compete for the area at the water surface with template molecules rather than be easily lifted. This was in fact the case—amphiphilic Au NPs (15% of OH groups at the ends of coating molecules) were found not to create any net-like structures in a matrix of phospholipids. We agree with such conclusion; however, our results presented in this article indicate that it is possible for amphiphilic Au NPs to form a net-like structure. We believe that the model presented in the aforementioned publication is still valid for our system, since we also observed lifting of the Au NPs from the water surface (see Fig. 5)—even despite the fact that the used Au NPs were amphiphilic. Moreover, similarly as in case of completely hydrophobic Au NPs, the Au NPs we used, having 10% of polar groups in the outer shell, tended to form rafts at the air–water interface (see Supporting Information). Such behavior indicated that the interactions between Au NPs were stronger comparing to interactions between Au NPs and the water surface (Sear et al. 1999; Imperio et al. 2008). Such statement raised a question: why Hassenkam and coworkers did not find the net-like structure in case of amphiphilic Au NPs and phospholipids mixtures?

It seems that the interactions between OH groups introduced to the outer shell of the Au NPs (15% of coating thiols) are fairly strong, comparing to the interactions between phospholipid molecules and the water surface. Therefore, the phospholipid could not act as a template and Au NPs were not lifted from the water surface during the compression of the Langmuir film. In our case, the balance between these interactions was different, as we used the 8CB for the matrix and only 10% of the thiols coating the Au NPs had a polar group attached at the end of the chain. We are planning to test this hypothesis in the future by using the Au NPs with different amounts of OH-terminated ligands under otherwise identical conditions.

In case of phospholipids, the 2D liquid phase decreases in size, whereas the 2D solid phase increases as the compression proceeds. The Au NPs are frozen out of the liquid phase and end up at the boundaries between the solid phase domains. The analysis of the isotherms of the films of mixtures of 8CB and Au NPs indicates a slightly different mechanism. The domains of multilayer stacks, and not a 2D solid monolayer, act as the condensed phase that causes the net-like structure formation. The structure of Au NPs in 8CB matrix appears upon film compression to more than 15 mN m^−1^. In case of compositions 1:6, 1:9, and 1:27 such value of surface pressure corresponds to a point at the second slope of the isotherm, behind the plateau. It is well known and proven that the plateau in the isotherm of 8CB corresponds to formation of a trilayer. In case of film of 1:9 composition, the observed surface coverage upon trilayer formation should equal 1:3 (8CB occupied thrice smaller area than in monolayer film), i.e., around 25% of the surface should be covered with Au NPs. However at π = 15 mN m^−1^, we observed the Au NPs coverage of around 13%, which corresponds to a coverage ratio around 1:7. Not all of the 8CB molecules formed densely packed trilayer domains in the observed film. This can be easily explained by the fact that the net-like structure was decompressed (from 18 mN m^−1^) prior to the transfer (at 15 mN m^−1^). As a result, a decrease in density of the packing of the molecules was observed. Therefore, the densely packed trilayer film should not be observed. Rather a coexistence of a trilayer and a monolayer film of 8CB as a matrix for the structure of Au NPs should be expected. The fitting of XRR profile indicated that the Au NPs were lifted from the surface by around 1.2 nm. Therefore Au NPs were placed on the monolayer film of 8CB and the particle-free cavities were filled with the trilayer domains of 8CB of rather loose compaction of molecules.

The Au NPs surface coverage in case of a sample of composition ratio 1:9 transferred at 18 mN m^−1^ reached a value of around 29% (see Fig. S7 Supporting Information). As was already mentioned, in case of formation of a trilayer film of 8CB, the Au NPs surface coverage should equal 25%. The observed, higher value indicates that the 8CB exhibits a layering transition to thicker films. Such behavior of 8CB was previously recognized (Xue et al. 1992). The second plateau at the π–*A* isotherm of 1:9 mixture is not visible because the Au NPs alter the compression properties of the trilayer-Au NPs composite film.

Overall, the process of formation of Au NPs net-like structures requires the initial compression of the film of Au NPs and 8CB mixture. During this process the trilayer film of 8CB is formed and Au NPs are “frozen out” and appear at the domains’ border. We found that the compression to around 18 mN m^−1^ is sufficient for this process to take place. When the initial compression was stopped at 15 mN m^−1^, the process took much time—an interval of 90 min was needed for the Au NPs to rearrange and appear at the domains’ border (see Fig. S8 in Supporting Information). This was probably caused by the difference in the compaction of the molecules—the higher the surface pressure was, the more condensed phase was formed at the air–water interface and the faster the “freezing-out” process was. The image analysis of the films of composition ratio 1:9 transferred at 15 mN m^−1^ with varying time interval prior to transfer (3 min to 90 min) revealed that the Au NPs surface coverage was almost constant (see Figs. 3d, f, S9). This confirmed 2D rearrangement (“freezing out”) of the Au NPs during the net-like structure formation due to condensed domains formation.

The decompression of the film of studied mixtures resulted in an increase of average size of the unit cell of the structure. This was due to a layering transition within the 8CB domains i.e. formation of a monolayer from thicker stacks. As a result, the 8CB molecules occupied a larger area. Due to the decompression of the multilayer stacks, the average area of unit cell could increase by factor smaller than 10 (depending on the number of layers in multilayer stacks). However, SEM and BAM observations revealed an increase of the unit cells from around 2200 μm^2^ (at 15 mN/m) to around 0.15 mm^2^ (at 0 mN/m). This implied an increase of the unit cell by a factor of around 70. Such effect was caused by further decompression of the 8CB monolayer to a 2D liquid phase. The process of unit cells growth stopped at some point (see Fig. 4)—probably when the 2D gaseous phase of 8CB appeared.

## Conclusions

This study presents a systematic investigation of self-assembly of amphiphilic Au NPs in a liquid crystalline matrix of 8CB at the air–water interface. The Langmuir films of mixtures of 8CB and Au NPs form a net-like structure, wherein Au NPs aggregate around circularly shaped Au NPs-free areas. The net-like patterns appear due to the initial compression to at least 18 mN m^−1^, as a result of formation of condensed phase of domains of a multilayer film of 8CB. The nanoparticles act as impurities in the liquid crystalline matrix and are “frozen out” to the edges of the LC domains. At lower surface pressures such process is very slow.

An analogous mechanism of formation of net-like patterns was reported previously for hydrophobic Au NPs. We confirmed its validity also in case of amphiphilic Au NPs (with 10% of charged ligands). The average size of the unit cell proved to be fully controllable during film decompression. The net-like pattern of desired average unit cell area could be easily and effectively transferred onto a solid substrate with use of the LB technique. SEM images indicated that the structures were uniform over large areas.

## Electronic supplementary material

Below is the link to the electronic supplementary material.

Supplementary material 1 (DOC 5066 kb)
