# Supplementary material for: Formation of net-like patterns of gold nanoparticles in liquid crystal matrix at the air–water interface
Source: J Nanopart Res. 2012 Mar 31;14(4):826. doi: 10.1007/s11051-012-0826-4 (PMC3394235; doi:10.1007/s11051-012-0826-4)
Supplement: Supplementary file 1 — Supplementary material 1 (DOC 5066 kb) [file 11051_2012_826_MOESM1_ESM.doc]

**SUPPORTING INFORMATION**

Formation of Net-like Patterns of Gold Nanoparticles in Liquid Crystal Matrix at the Air-Water Interface

Jan Paczesny^a^, Krzysztof Sozański^a^, Igor Dzięcielewski^b^, Andrzej Żywociński^a^, Robert Hołyst^*,a^

^a^ Institute of Physical Chemistry, Polish Academy of Sciences, Kasprzaka 44/52, 01-224 Warsaw, Poland.

^b^ Institute of High Pressure Physics Unipress, Polish Academy of Sciences, Sokołowska 29/37, 01-142 Warsaw, Poland.

jpaczesny@ichf.edu.pl, krzysiek.sozanski@gmail.com, igor@unipress.waw.pl, azywocinski@ichf.edu.pl, rholyst@ichf.edu.pl

*Robert Hołyst

Fax: 48 22343 3333; Tel: 48 223433102, Email: [rholyst@ichf.edu.pl](mailto:rholyst@ichf.edu.pl) or [robert.holyst@gmail.com](mailto:robert.holyst@gmail.com)


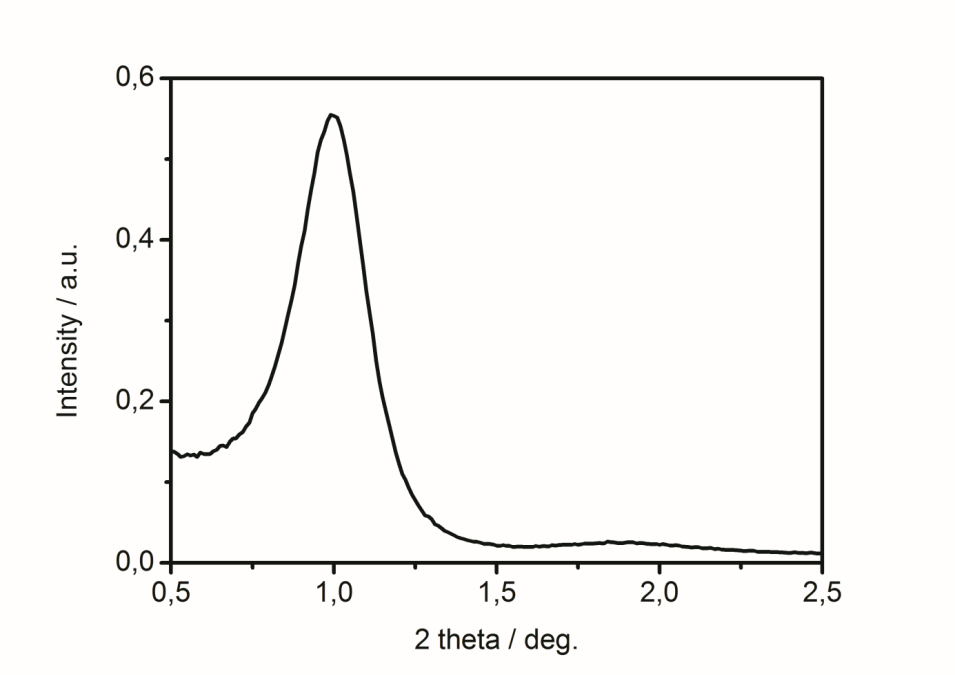


**Figure S1** Small Angle X-rays Scattering (SAXS) measurement for pure Au NPs. The size of the particle was obtained from fitting, and it equals 8.9 nm.


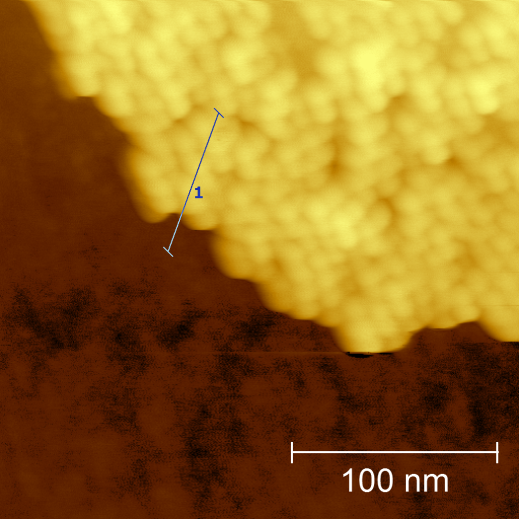


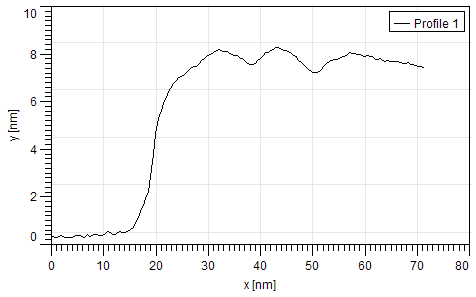


**Figure S2** Confirmation of the size of the Au NPs with use of atomic force microscopy (AFM). The obtained value is in good agreement with the SAXS results.

**
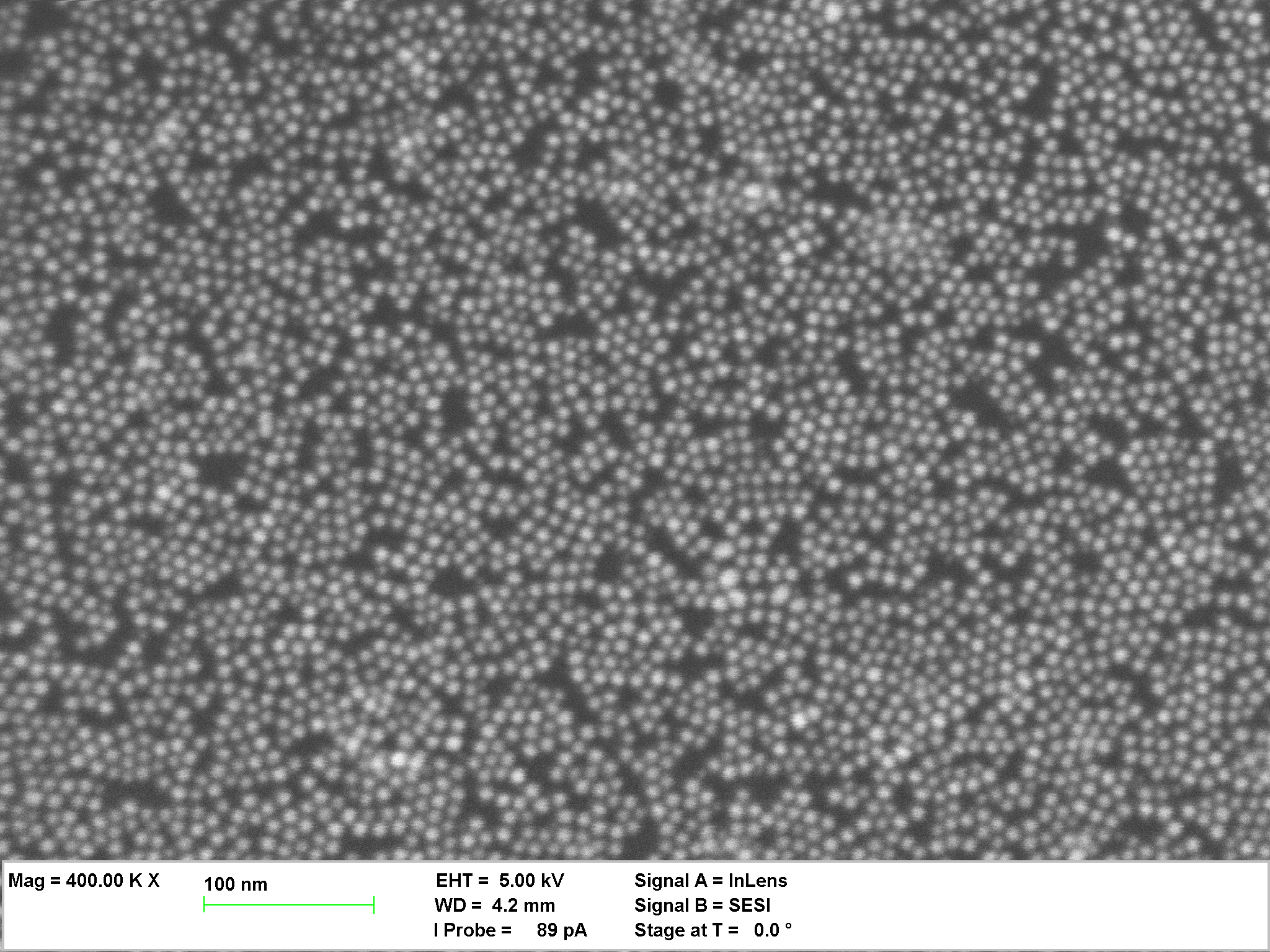
**
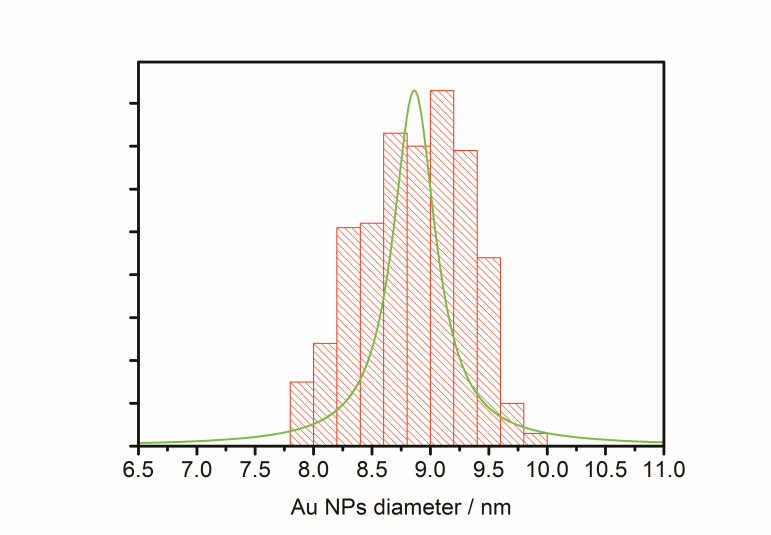


**Figure S3** High resolution SEM pictures additionally confirm narrow size distribution of the Au NPs.


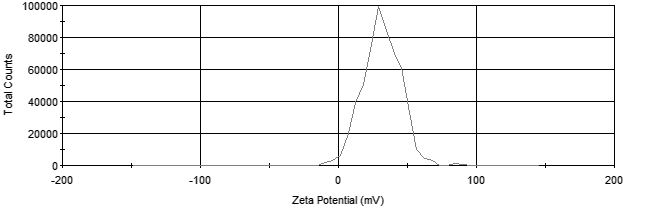


**Figure S4** Positive value of zeta potential confirmed the amphiphilic character of Au NPs. At the ends of the 10% of thiol chains the polar group was introduced to ensure increased hydrophilicity of the particles.

## Details on descriptions of concentration ratios used throughout the article

When known volume of solution of pure 8CB or Au NPs was spread on the water surface and compressed, the collapse point (kink at the isotherm) indicated the point corresponding to a dense monolayer. In other words, the area between the barriers at the moment of film collapse is equal to the maximal area that can be covered by a dense monolayer with the used amount of solution. If the volume of applied solution is divided by the collapse point area, the result is the aforementioned concentration (which can be expressed in μl/cm^2^). Relation between amounts of Au NPs and 8CB in mixed solutions is denoted throughout the article as a ratio of such concentrations. Proper amounts of both compounds were found in LB experiments, since it is difficult to determine the absolute concentration of gold nanoparticles with required accuracy.

See also Figure S5.


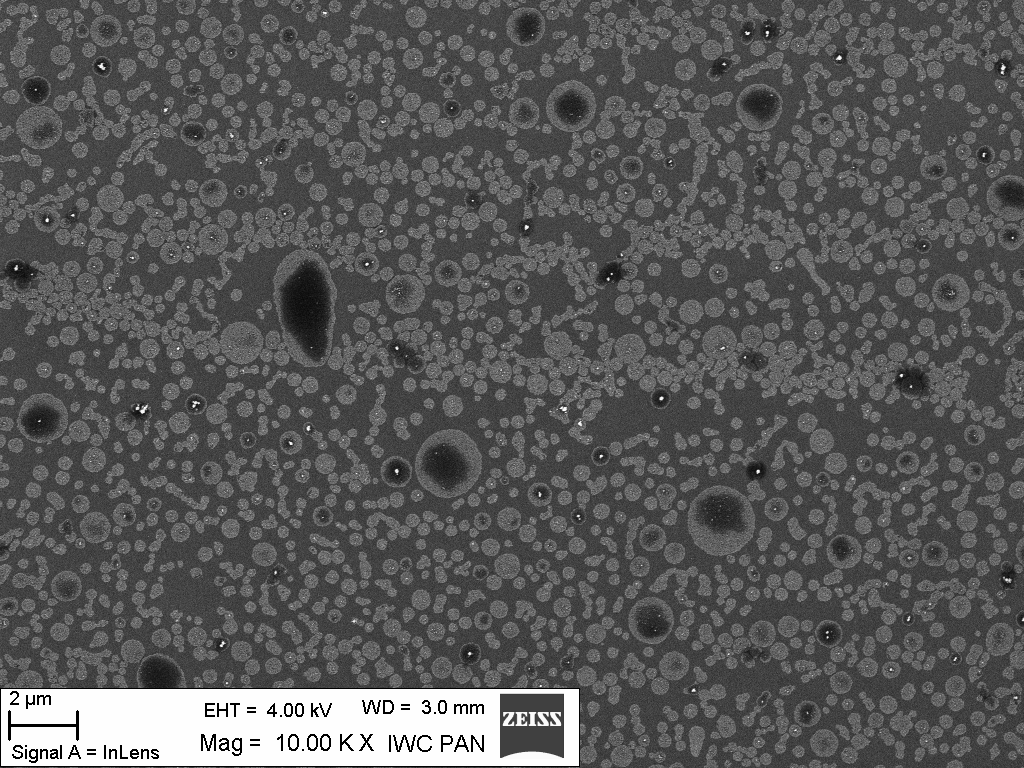

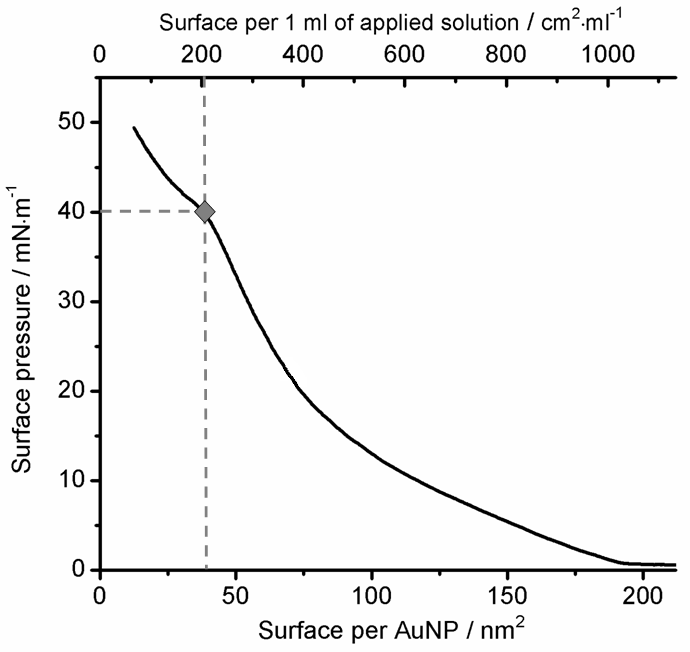


**Figure S5** The π(A) isotherm of the used Au NPs. SEM image of film transferred at 30 mN m^-1^ revealed formation of circular domains at the air/water interface. The marked point around 40 mN m^-1^ corresponds to a dense monolayer.


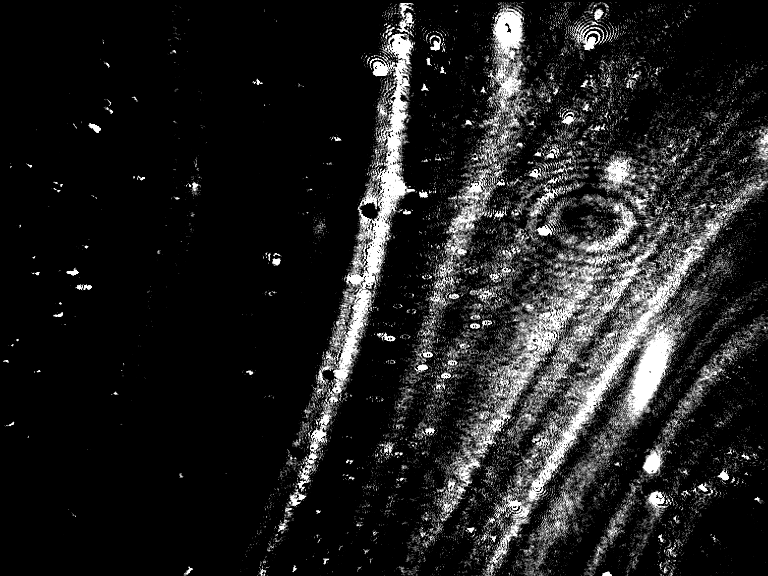


**Figure S6** BAM image of a film of pure Au NPs, taken at around 5 mN m^-1^.


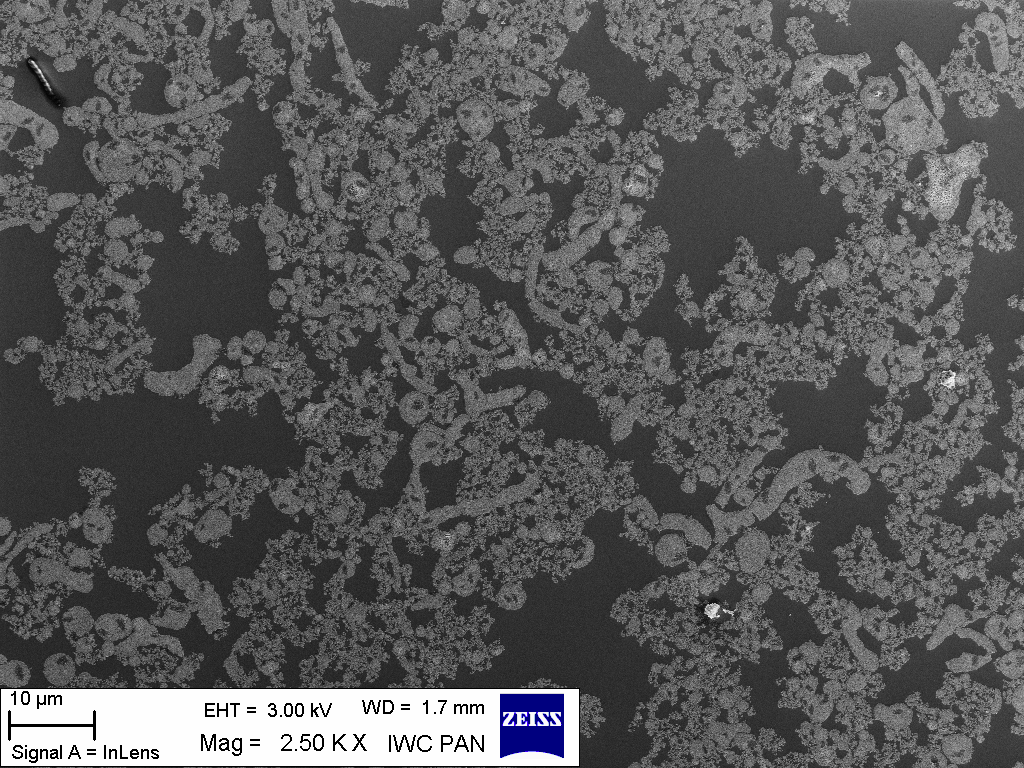


**Figure S7** SEM image of a film transferred at 18 mN m^-1^ with no time interval after reaching the target surface pressure. The image analysis indicates a surface coverage by the Au NPs of around 29%.


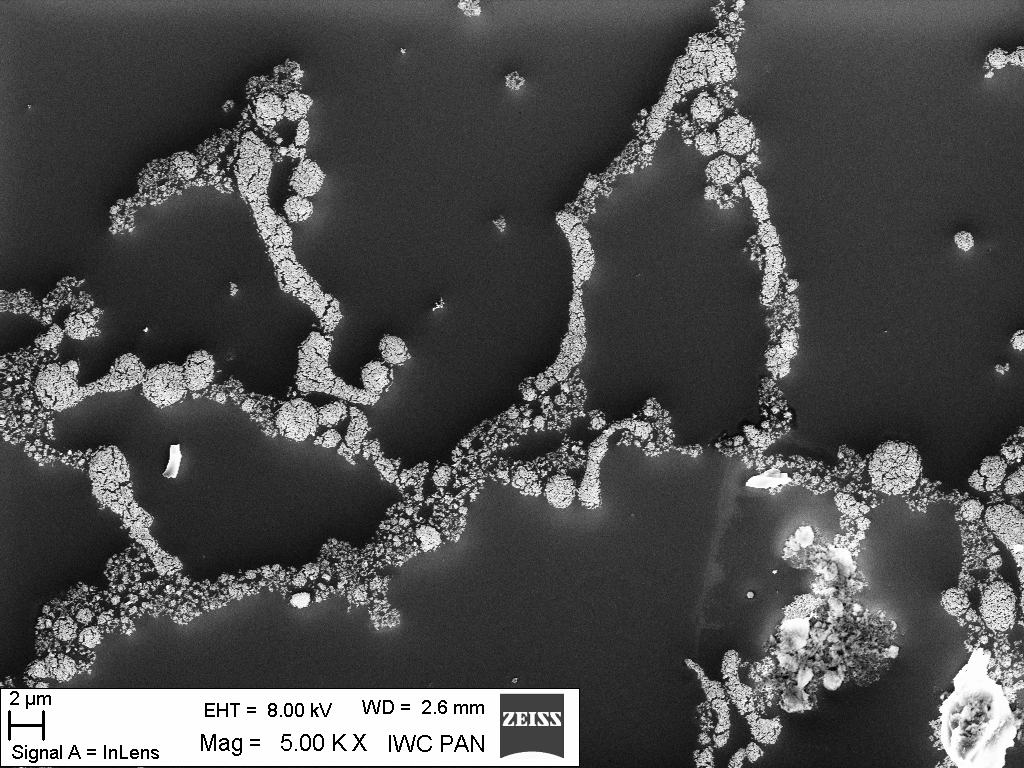


**Figure S8** SEM image of Au NPs network after removal of organic compounds with use of NaBH_4_.


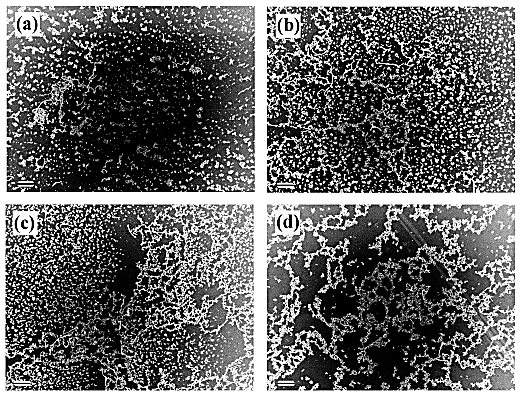


**Figure S9** SEM images of films of 1:9 composition transferred after a) 10 min, b) 30 min, c) 60 min, d) 90 min of conditioning at a surface pressure equal 15 mN m^-1^; scale bar 20 μm.
